# Better Longitudinal Adherence to Antiretroviral Therapy Among Virally Suppressed People With HIV Is Associated With Reduced Occurrence of Serious Non-AIDS Events

**DOI:** 10.1093/ofid/ofag196

**Published:** 2026-04-03

**Authors:** Roland van Rensburg, Mercy Rop, Daniel Mashishi, Innocent Maposa, Katherine Tassiopoulos, Kunling Wu, Jose R Castillo-Mancilla, Netanya S Utay, Kristine M Erlandson, Eric H Decloedt

**Affiliations:** Division of Clinical Pharmacology, Department of Medicine, Faculty of Medicine and Health Sciences, Stellenbosch University, Cape Town, South Africa; Division of Epidemiology and Biostatistics, Department of Global Health, Faculty of Medicine and Health Sciences, Stellenbosch University, Cape Town, South Africa; Division of Epidemiology and Biostatistics, Department of Global Health, Faculty of Medicine and Health Sciences, Stellenbosch University, Cape Town, South Africa; Division of Epidemiology and Biostatistics, Department of Global Health, Faculty of Medicine and Health Sciences, Stellenbosch University, Cape Town, South Africa; Center for Biostatistics in AIDS Research, Harvard T. H. Chan School of Public Health, Boston, Massachusetts, USA; Center for Biostatistics in AIDS Research, Harvard T. H. Chan School of Public Health, Boston, Massachusetts, USA; Division of Infectious Diseases, University of Colorado Anschutz Medical Campus, Aurora, Colorado, USA; Department of Internal Medicine, University of Texas Southwestern Medical Center, Dallas, Texas, USA; Division of Infectious Diseases, University of Colorado Anschutz Medical Campus, Aurora, Colorado, USA; Division of Clinical Pharmacology, Department of Medicine, Faculty of Medicine and Health Sciences, Stellenbosch University, Cape Town, South Africa

**Keywords:** adherence, antiretroviral therapy, cardiovascular, death, serious non-AIDS events

## Abstract

**Background:**

Despite effective antiretroviral therapy (ART), serious non-AIDS events (SNAEs), including cardiovascular disease and death, remain prevalent among people with HIV (PWH). Imperfect ART adherence despite viral suppression may contribute to this risk, underscoring the need to clarify the relationship between adherence, viral load dynamics, and SNAE development.

**Methods:**

We performed an analysis of deidentified data of participants in the prospective observational Advancing Clinical Therapeutics Globally for HIV/AIDS and Other Infections (ACTG) A5001 and A5322 studies. The baseline was defined as the time at the first viral suppression to <200 copies/mL after treatment initiation. Antiretroviral therapy adherence was assessed using the periodically administered ACTG self-report questionnaire and dichotomized as >90% versus ≤90% adherence. Data were primarily analyzed using inverse probability of censoring weights modeling.

**Results:**

Among 2940 participants followed for a median of 6.2 years, 237 SNAEs occurred, comprising 103 deaths and 134 cardiovascular events. Nearly all participants (95.9%) remained virally suppressed to <200 copies/mL over the full duration of follow-up. Adherence to ART >90% showed a protective effect on the development of all SNAEs: adjusted hazard ratio (aHR) 0.612 (95% confidence interval .390–.961). When only the development of the first SNAE was considered, the association remained substantially similar: aHR 0.641 (95% confidence interval .400–1.027).

**Conclusions:**

Adherence to ART >90% compared to ≤90% reduces SNAE risk even in virally suppressed PWH, underscoring the need for continued adherence support to improve long-term health outcomes for PWH.

## Teaser

Our analysis of 2940 virally suppressed people with HIV showed that higher antiretroviral therapy adherence significantly reduced the serious non-AIDS events of cardiovascular disease and death. Adherence >90% showed a 38.8% reduced hazard after adjusting for confounding covariates, emphasizing adherence's importance for long-term health outcomes.

Since the inception of highly active antiretroviral therapy (ART), HIV-associated communicable diseases and AIDS-related events have decreased significantly due to viral suppression and immune restoration [1]. However, the incidence of HIV-associated noncommunicable diseases, in particular serious non-AIDS events (SNAEs), is steadily increasing [2–5]. Serious non-AIDS events are significant non-AIDS-defining health complications in people with HIV (PWH), including liver and kidney disease, malignancies, and importantly, cardiovascular disease (CVD) and death [5]. People with HIV on ART present with SNAEs earlier and with fewer traditional risk factors compared to their counterparts without HIV, likely reflecting accelerated immune activation and aging [6–8]. A large meta-analysis showed that the global burden of HIV-associated CVD tripled over the last 20 years, despite advances in ART and adherence strategies [9]. The mechanism is likely multifactorial but has been strongly linked to persistent HIV-associated inflammation [10, 11].

Increasing focus is being placed on addressing modifiable risk factors for inflammation beyond viral suppression. The REPRIEVE trial showed that pitavastatin reduced major adverse cardiovascular events in PWH, but this appeared to be driven primarily by the reduction in low-density lipoprotein cholesterol without clear beneficial effect on inflammatory biomarkers [12]. Accordingly, optimization of both traditional and HIV-specific risk factors is required, and enhancing ART adherence has emerged as an important modifiable factor [13, 14]. An analysis of the Swiss HIV Cohort Study found that missing ≥2 doses in the last 4 weeks was significantly associated with increased noncardiovascular-related mortality in virally suppressed PWH: hazard ratio 2.21 (95% confidence interval [CI] 1.37–3.57) [15]. Similarly, the Multicenter AIDS Cohort Study (MACS) found that virally suppressed men with <100% self-reported adherence had significantly greater coronary artery stenosis progression than perfectly (100%) adherent men over 4.5 years [16]. Therefore, while imperfect adherence may result in clinically relevant viral suppression to limit transmission, low-level viremia (LLV), defined as 50–1000 copies/mL, may persist especially with older ART regimens with deleterious health consequences such as SNAEs [17, 18].

The impact of LLV on SNAE development was shown in a recent study by Ganesan et al [19]. Using data from the US Military HIV Natural History study, they found that SNAE development was significantly associated with the lower range of LLV (50–200 copies/mL) among the 2528 participants over 8.2 years: hazard ratio 1.3 (95% CI 1.2–1.4) compared to consistent suppression <50 copies/mL. However, participants were categorized only according to the highest viremia stratum achieved, and SNAEs remained prevalent in those classified as virally suppressed (11.5% vs 14.5% in the LLV group) [19]. Furthermore, a Swedish nationwide observational cohort study attempted to control for the time effect by adding viremia-time as a covariate but also analyzed participants only by highest viremia category [4]. Given that suboptimal adherence is common in real-world practice [20], the time-varying effect of imperfect ART adherence on SNAE development in virally suppressed PWH remains uncertain.

In view of the above, there is a need to identify factors beyond viral suppression and traditional risk factors that can be targeted to reduce SNAEs [14]. Imperfect ART adherence, although sufficient to suppress HIV RNA to <50 copies/mL, may represent such an underrecognized, highly prevalent risk factor, especially when viral load is used as a surrogate for adherence [14, 21–23]. Given the significant burden and seriousness of the SNAEs involving CVD and mortality, our study sought to determine the time-varying effect of self-reported ART adherence and HIV RNA levels on SNAE incidence (CVD and death) in PWH who are virally suppressed at baseline.

## METHODS

### Population

We performed an analysis of deidentified data of participants that were enrolled in the prospective observational Advancing Clinical Therapeutics Globally for HIV/AIDS and Other Infections (ACTG) A5001 (NCT00001137) and A5322 studies. The methodologies of these studies have been previously described [24, 25]. In short, A5001 prospectively enrolled a cohort of 5972 PWH; 4748 had initiated ART through an ACTG clinical trial (“parent study”) and were ART-naive, and 1224 were treatment-experienced at parent study entry. The goal of A5001 was to assess various longitudinal outcomes between 2000 and 2013. Selected older participants from A5001 (ART-naïve at parent study entry, age ≥40 years, n = 1035) were enrolled into the subsequent A5322 study that examined further long-term health outcomes between 2013 and 2021.

### Study Design

We conducted a prospective, longitudinal analysis over the maximum duration of follow-up for each participant on A5001 and A5322. Our aim was to determine the time-varying effect of annual self-reported ART adherence using the ACTG adherence questionnaire with SNAEs development. Eligibility criteria for this analysis included being ART-naive at parent study entry and without study-defined SNAEs at baseline. Baseline was defined as the time of the first viral suppression, specified as <200 copies/mL, after treatment initiation. Both A5001 and A5322 studies were approved by each participating sites’ institutional review boards and all participants provided written, informed consent.

### Variables

The primary exposure variable was adherence to ART, which was captured in both parent studies using the ACTG Adherence Self-Report form. Participants were asked to indicate how many doses were missed over the preceding 4 days. A derived adherence percentage was calculated by dividing the number of taken doses over the preceding 4 days by the number of prescribed doses over the preceding 4 days (Supplementary Equation 1). Mean adherence was then dichotomized into ≤90% and >90% categories, in keeping with general consensus for optimal adherence thresholds [26]. The development of a SNAE over the course of follow-up was defined as (1) death due to a non-AIDS event or (2) CVD events such as acute coronary syndrome and stroke or transient ischemic attack. “All SNAEs” as the primary outcome included all SNAE events over a participant's follow-up, whereas “first SNAE” in the sensitivity analysis was defined as the first recorded SNAE event for a participant. Transient viral blips (200 to <500 copies/mL) during follow-up for first SNAE were allowed if a subsequent measurement returned to <200 copies/mL [27], and viral loads ≥200 copies/mL were allowed for subsequent SNAEs (all SNAE analysis). Serious non-AIDS events and deaths were ascertained from the available documentation or medical records to the study sites and reviewed by protocol clinicians who referred to standardized diagnosis appendices developed by the ACTG data management center. All study variables were used as collected from the primary studies.

### Statistical Approach

Baseline sociodemographic and clinical characteristics were summarized using descriptive statistics. Continuous variables were reported as means with standard deviations or medians with interquartile ranges (IQRs), as appropriate, and categorical variables as counts and percentages. To address potential bias from dependent censoring and estimate the effect of time-varying ART adherence on SNAE development over follow-up, we applied inverse probability of censoring weighting (IPCW). The weights were derived from a censoring model estimating the probability of remaining uncensored using a cluster-adjusted Cox proportional hazards model with baseline and time-varying covariates, including age, sex, viral load, any comorbidities, body mass index, systolic blood pressure, smoking status, protease inhibitor (PI)–based ART regimen, CD4 cell count, and nadir CD4 cell count. These variables were selected for their known association with CVD risk and SNAEs (including PI-based regimens [28]) and specifically for confounding control. The inverse probabilities served as weights to adjust for differential censoring.

As a sensitivity analysis, we evaluated the association of adherence with first SNAEs using IPCW, as well as adherence on first and all SNAE development using generalized estimating equations (GEEs). Additional sensitivity analyses were performed for first and all SNAEs by restricting participants to those who remained virally suppressed to <50 copies/mL for the duration of follow-up.

Missing time-varying covariate values were imputed using the last observation carried forward method, assuming stability since the previous visit. All analyses were subsequently performed using complete-case analysis. All analyses were conducted using R version 4.4.3 (R Core Team 2025). A *P*-value <.05 was considered statistically significant.

## RESULTS

The ACTG A5001 and A5322 datasets included 2940 unique participants, with study visits spanning over 23 years from 1998 to 2021. The included cohort were mostly young (mean age 39 years) and male (82%), with the majority receiving nonnucleoside reverse transcriptase inhibitor (NNRTI)– or PI-based ART at baseline (Table 1). Thirty-eight percent had at least one comorbid diagnosis of hypertension, dyslipidemia, or diabetes, with all participants being on treatment for the comorbidities at baseline (Supplementary Table 1). One third (32%) were current smokers at study baseline.

**Table 1.** Baseline Characteristics

Characteristics	Overall (n = 2940)
Age (years)	
Median (IQR)	39.4 (32.0–46.6)
Sex	
Female	518 (18%)
Male	2422 (82%)
Race/Ethnicity	
White non-Hispanic	1347 (46%)
Black non-Hispanic	857 (29%)
Hispanic (regardless of race)	648 (22%)
Other	82 (2.8%)
Missing	6 (0.2%)
BMI (kg/m^2^)	
Median (IQR)	25.3 (22.6–28.5)
Missing	4 (0.1%)
ART class (backbone regimen)	
NNRTIs	1243 (42.3%)
PI	1218 (41.4%)
INSTI	349 (11.8%)
Other	130 (4.4%)
Hepatitis serology^a^	
Negative	79 (2.7%)
Positive	12 (0.4%)
Missing	2849 (96.9%)
Comorbidities^b^	
Hypertension	850 (28.9%)
Dyslipidemia	552 (18.7%)
Diabetes	216 (7.3%)
Systolic blood pressure (mmHg)	
Median (IQR)	120 (110–130)
Physical activity^c^	
<3 d of both vigorous and moderate activities	299 (10.2%)
≥3 d of either vigorous or moderate activities	352 (12%)
Missing	2289 (77.9%)
Smoking status	
Current	948 (32%)
Never	1327 (45%)
Prior	646 (22%)
Missing	19 (0.6%)
Substance use (excluding tobacco)	
Current	133 (4.5%)
Never	312 (10.6%)
Prior	207 (7%)
Missing	2288 (77.8%)
HIV-1 RNA (copies/mL)	
Median (IQR)	<50 (<40 to <50)
CD4 cell count, cells/µL	
Median (IQR)	397 (258–549)
Nadir, median (IQR)	222 (87–323)

Abbreviations: ART, antiretroviral therapy; BMI, body mass index; INSTI, integrase strand transfer inhibitor; HBc Ab, hepatitis B virus core antibody; HBs AG, hepatitis B virus surface antigen; HCV Ab, hepatitis C virus antibody; IPAQ, International Physical Activity Questionnaire; IQR, interquartile range; NNRTI, nonnucleoside reverse transcriptase inhibitor; PI, protease inhibitor.

^a^Hepatitis serology includes any positive result for HBc Ab, HBs Ag, or HCV Ab.

^b^Participants may have had more than one comorbidity, therefore counts are not mutually exclusive. Details of comorbid conditions are listed in Supplementary Digital Content 1.

^c^Assessed using the IPAQ.

Participants were followed from baseline for a median of 6.2 years (IQR 3.1–10.5). For the duration of follow-up, median viral load was suppressed to <50 copies/mL (IQR <50–62) over an average of 3.1 sampling timepoints per year (IQR 2.6–3.6). The viral load range spanned from <20 61 922 copies/mL, although all values >200 copies/mL occurred after the first SNAE (n = 121). A total of 237 SNAE events occurred (8.1% of total population): 103 deaths and 134 nonfatal CVD-related events (Table 2). Twenty-six deaths (20.6%) were due to a CVD cause. The median time to first SNAE event was 5.0 years (IQR 2.6–9.4). Of those who experienced a SNAE, most developed only one over the duration of follow-up (median 1 [IQR 1–1; range 1–5]). Derived self-reported ART adherence using the ACTG questionnaire was high, with mean adherence of 97.1% (95% CI 96.9–97.2) captured over a median of 2.2 reports per year (IQR 1.8–3.0) over the follow-up period.

**Table 2.** Serious Non-AIDS Event Outcomes

SNAE	N = 237
Death	103 (43.5)
Cardiovascular (including stroke)	26 (25.2)
Cancer	31 (30.1)
Respiratory	11 (10.7)
Infection/sepsis	8 (7.8)
Renal/metabolic/endocrine	6 (5.8)
Liver/gastrointestinal	3 (2.9)
Multisystem	1 (1)
Unknown	17 (16.5)
Cardiovascular disease	134 (56.5)
Acute coronary syndrome	34 (25.4)
Stroke or transient ischemic attack	32 (23.9)
Venous thromboembolism	19 (14.2)
Coronary artery disease	22 (16.4)
Arrhythmia	13 (9.7)
Congestive heart failure	7 (5.2)
Angina pectoris	4 (3.0)
Peripheral artery disease	3 (2.2)

Data presented as n (%).

Abbreviation: SNAE, serious non-AIDS event.

**Figure 1.**
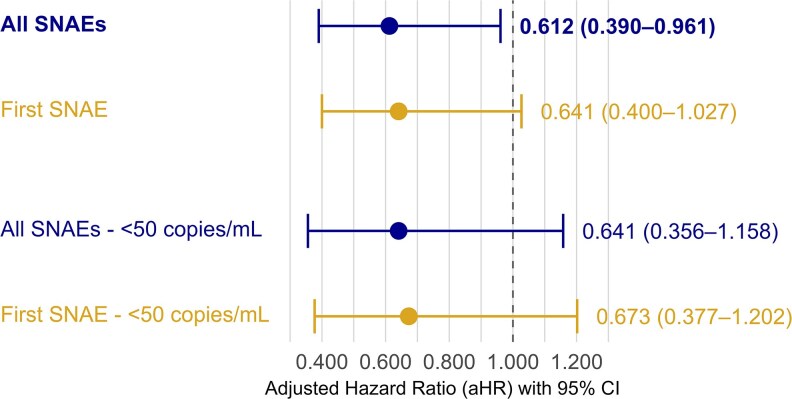
Adjusted hazard ratios (aHRs) with 95% confidence intervals (CIs) for associations of adherence with serious non-AIDS events (SNAEs) in the primary and sensitivity analyses, estimated using inverse probability of censoring weights. The dashed vertical line represents the null value (aHR = 1). Horizontal bars indicate 95% CIs, and points represent adjusted point estimates. An aHR >1 indicates increased risk, and an aHR <1 indicates reduced risk of SNAEs.

Adherence to ART >90% showed a protective effect on the development of all SNAEs over follow-up under IPCW: adjusted hazard ratio (aHR) 0.612 (95% CI .390–.961) (Figure 1; Supplementary Table 2), translating to a 38.8% reduced hazard after adjusting for confounding covariates. When only first SNAEs were considered, the association with adherence >90% remained protective with a similar hazard reduction: aHR 0.641 (95% CI .400–1.027) (Figure 1; Supplementary Table 3).

### Sensitivity Analyses

The GEE results demonstrated the validity of ART adherence >90% as a protective factor against the risk of developing first SNAE (Supplementary Table 4) and marginally for all SNAEs (Supplementary Table 5): odds ratio (OR) 0.141 (95% CI .025–.803) and OR 0.189 (95% CI .028–1.271), respectively. The GEE results suggested some model instability particularly with respect to smoking status and PI-based regimens, likely due to the lack of adjustment for missingness and censoring, whereas the clustered-adjusted Cox model under IPCW accounted for missingness and censoring.

Results from a further analysis restricted to only those who remained virally suppressed to <50 copies/mL for the duration of follow-up were largely similar for all SNAEs (aHR 0.641, 95% CI .356–1.158) and first SNAE using IPCW (aHR 0.673, 95% CI .377–1.202), although not statistically significant (Figure 1; Supplementary Tables 6 and 7).

## DISCUSSION

Our analysis of the ACTG A5001 and A5322 studies with virally suppressed PWH found a significant 38.8% reduction in the hazard of the major SNAE events of death or CVD with ART adherence >90% compared to ≤90%. The findings remained largely similar in the sensitivity analyses considering only the first SNAE event or restricting the analysis to participants who remained suppressed <50 copies/mL during follow-up, supporting the primary outcome's robustness. Our findings add to the growing body of evidence to look beyond viral suppression for improved long-term health benefits in PWH.

Our study adds a distinct perspective by focusing on SNAE outcomes in virally suppressed PWH, as previous longitudinal observational studies compared virally suppressed to viremic participants. Illustratively, the study by Ganesan et al [19] found a significantly increased hazard for SNAE development (HR 1.3, 95% CI 1.2–1.4) for LLV versus suppression, increasing to 1.6 (95% CI 1.5–1.7) for 200–999 copies/mL and 1.7 (95% CI 1.7–1.8) for viral failure (≥200 copies/mL on ≥2 successive occasions or ≥1000 copies/mL once). The Swedish InfCareHIV register study similarly found an aHR of 2.0 (95% CI 1.2–3.6) for higher LLV versus suppression over 49 986 person-years [4]. However, both studies analyzed participants according to the highest viremia stratum, leaving uncertainty about SNAE risk in consistently suppressed PWH and how varying adherence influences SNAE outcomes.

Castillo-Mancilla et al [29] aimed to address this gap with modeling work. Using data from 3 randomized clinical trials, they linked improved adherence to reduced inflammatory (interleukin-6) and coagulopathy (D-dimer) biomarkers. The model estimated that increasing adherence by 10% (from 90% to 100%) could reduce SNAE risk by 6%–21% and up to 37% for a 20% increase in adherence. Our observed 39.5% reduction aligns closely with this projection. Building on this work, our study used hard clinical outcomes (death or CVD) with viral load as a continuous, time-varying measure, with 67.5% remaining virally suppressed to <50 copies/mL over the full duration of follow-up (and 95.9% to <200 copies/mL).

While Castillo-Mancilla et al linked imperfect adherence and SNAEs to increased inflammation in suppressed PWH, the causal mechanisms remain unclear. In viremic participants, studies have postulated that ongoing viral replication drives immune activation and persistent inflammation, leading to SNAE development [4, 19]. In virally suppressed PWH, the drivers may be more nuanced, although still likely related to immune activation and persistent inflammation. Possible mechanisms in suppressed PWH are therefore plausibly linked to imperfect ART adherence, and include, first, viral blips or intermittent viremia between viral load measurements, which are often only done once or twice a year in clinical practice; second, replication-incompetent proviruses that still produces viral proteins and extracellular virus-like particles; third, viral replication below the commercial assays’ detection limit; or fourth, tissue-level replication of competent viruses from the viral reservoir, but due to mostly suppressive ART, multiple local abortive cycles are effected that are sufficient to create inflammation but not evolution [30, 31].

The clinical implications of residual viremia or replication-incompetent proviruses remain contested [15], but regardless of the mechanism, persistent inflammation appears to be central [5]. Conversely, normalization of inflammation with improved adherence was shown in the MACS, where suppressed men (<50 copies/mL) with 100% adherence had proinflammatory biomarker concentrations (interleukin-6, interleukin-6 receptor, interleukin-1β, and interferon-γ) comparable to men without HIV [32], suggesting that consistently high adherence may normalize inflammation and SNAE risk. Interestingly, open-label trials of less-than-daily ART showed no inflammatory biomarker increase, but included highly selected, long-term suppressed participants with short follow-up, limiting generalizability [33, 34]. Furthermore, factors related to socioeconomic challenges and health literacy that are not often measured in trials could impact both lower ART adherence and risk of SNAE [35, 36].

Despite remaining questions, the evidence supports an “adherence continuum,” whereby higher degrees of ART adherence continue to confer additional clinical benefit even after reaching the threshold for modern viral suppression. This is likely rooted in improved adherence minimizing transient blips or LLV not captured by standard viral load monitoring. This underscores the need for rigorous adherence counseling, not only to prevent viral failure or LLV but also to reduce the long-term burden of SNAEs. Furthermore, PWH on suppressive ART are considered low-risk for HIV transmission and HIV-associated communicable diseases and are therefore a population that is often overlooked in ART programs for interventions that improve morbidity and mortality. While modern ART regimens’ pharmacokinetic and high genetic barrier characteristics mostly allow for missed doses without immediate virological penalty, this tolerance does not appear to protect against chronic inflammation driving SNAEs. Therefore, clinical guidelines and counseling should emphasize adherence for long-term health maintenance and comorbidity prevention and not solely for viral suppression or transmission control. Enhanced ART adherence may also improve adherence to other chronic medications, such as antihypertensives and statins.

The strengths of this study include the large, longitudinal dataset and the robust IPCW approach, which effectively adjusts for potential bias arising from dependent censoring. By modeling the probability of remaining uncensored throughout the study period, IPCW adjusts the observed data to account for individuals lost to follow-up, facilitating valid estimation of time-varying adherence effects under the assumption that censoring is conditionally independent of the outcome given the observed covariates. We also add a distinct perspective to the existing literature by only considering virally suppressed participants at baseline, using hard outcomes of CVD or death, and modeling viral load as a continuous variable.

Limitations of our study include, first, an average of 3 viral load measurements per year, and subsequently viral blips or LLV between study visits cannot be excluded. However, since long-term suppressed PWH often have viral loads checked only once per year [15, 29], our findings highlight the value of stronger adherence counseling given this real-world sparse testing. Second, the study population reflects an older global north setting, and may not be representative of the current majority of PWH globally who tend to be younger, female, and mostly on integrase strand transfer inhibitor (INSTI)–based ART. Third, high PI use may have confounded CVD risk [28], although we included PI-based regimen as a confounding variable in all of the models and found no significant difference in outcomes between PI-based and other regimens (χ^2^ 2.63, *P* = .105). Moreover, newer evidence also links CVD risk to modern INSTI regimens [37].

As the field of ART continues to evolve, use of long-acting injectable and oral ART are likely to increase. While cumulative data show noninferiority of long-acting ART compared to daily oral ART for viral suppression, concerns remain regarding durable efficacy considering the potential for LLV during the tail end of the dosing interval [38]. If suppression remains durable, further research should assess inflammatory biomarker trends and SNAE risk during the elimination phase of drug concentrations. Future studies should also consider larger prospective trials with robust adherence measures (eg, intracellular tenofovir diphosphate concentrations) to directly investigate the effect of improved adherence on SNAE reduction.

In conclusion, our study strengthens the evidence for enhanced efforts to improve adherence even in virally suppressed PWH due to its association with reduced SNAE risk. Globally, adherence remains challenging due to structural barriers to care, competing priorities, or pill and healthcare fatigue. HIV programs and healthcare workers should continue to emphasize optimal adherence beyond viral suppression, as the long-term health benefits of suppression may be attenuated without addressing underlying inflammation and immune activation.

## Supplementary Material

ofag196_Supplementary_Data

## Supplementary Data

Supplementary materials are available at *Open Forum Infectious Diseases* online. Consisting of data provided by the authors to benefit the reader, the posted materials are not copyedited and are the sole responsibility of the authors, so questions or comments should be addressed to the corresponding author.
