# Supplementary material for: Better Longitudinal Adherence to Antiretroviral Therapy Among Virally Suppressed People With HIV Is Associated With Reduced Occurrence of Serious Non-AIDS Events
Source: Open Forum Infect Dis. 2026 Apr 3;13(4):ofag196. doi: 10.1093/ofid/ofag196 (PMC13082379; doi:10.1093/ofid/ofag196)
Supplement: ofag196_Supplementary_Data [file ofag196_supplementary_data.docx]

**Better Longitudinal Adherence to Antiretroviral Therapy Among Virally Suppressed People with HIV is Associated with Reduced Occurrence of Serious Non-AIDS Events**

Van Rensburg R, et al

**SUPPLEMENTAL DIGITAL CONTENT 1**

**Equation 1: Derived adherence percentage calculation**

The data sets reported adherence as “Total doses missed over the preceding 4 days” related to “Prescribed doses per day”. We adapted the data to derive positive adherence, i.e. taking the doses compared to missing them:

$Adherence \left( \% \right)=\frac{Total number of doses taken over the preceding 4 days}{Total number of prescribed doses over the preceding 4 days}\times100$

**ADDITIONAL BASELINE CHARACTERISTICS**

**Table 1: Comorbidities**

| **Condition(s)** | **n (%)** |
| --- | --- |
| Hypertension only | 468 (16) |
| Hypertension + Dyslipidemia | 238 (8.1) |
| Dyslipidemia only | 204 (7) |
| Diabetes + Dyslipidemia + Hypertension | 74 (2.5) |
| Hypertension + Diabetes | 70 (2.4) |
| Diabetes only | 36 (1.2) |
| Diabetes + Dyslipidemia | 36 (1.2) |
| None | 1,806 (61.6) |

N=2,940

**PRIMARY ANALYSIS – ASSOCIATION OF ADHERENCE WITH SNAEs**

**Table 2: Adjusted associations with all SNAEs using inverse probability of censoring weights**

| **Characteristic*** | **aHR** | **Robust se** | **P-value** | **95% CI** |
| --- | --- | --- | --- | --- |
| **ADHERENCE LEVEL, >90%** | **0.612** | **0.230** | **0.033** | **(0.390 - 0.961)** |
| Age, years | 1.005 | 0.008 | 0.558 | (0.988 - 1.021) |
| Sex (Ref: Female) | 1 |  |  |  |
| Male | 1.391 | 0.228 | 0.147 | (0.891 - 2.174) |
| HIV viral load, log copies/mL | 1.183 | 0.125 | 0.181 | (0.925 - 1.512) |
| Any comorbidities, yes | **1.795** | **0.194** | **0.003** | **(1.228 - 2.623)** |
| Body mass index (BMI), kg/m^2^ | 0.994 | 0.019 | 0.731 | (0.958 - 1.031) |
| Systolic blood pressure (mmHg) | 0.998 | 0.005 | 0.750 | (0.988 - 1.009) |
| Smoking status (Ref: Current) | 1 |  |  |  |
| Never smoked | **0.337** | **0.202** | **<0.001** | **(0.227 - 0.500)** |
| Former smoker | **0.423** | **0.182** | **<0.001** | **(0.296 - 0.605)** |
| Protease inhibitor (PI)-based regimen (Ref: No) | 1 |  |  |  |
| Yes | **1.448** | **0.188** | **0.048** | **(1.002 - 2.092)** |
| CD4 cell count, cells/µL | **0.998** | **0.001** | **0.002** | **(0.997 - 0.999)** |
| Nadir CD4 cell count, cells/µL | 1.000 | 0.001 | 0.749 | (0.999 - 1.001) |

aHR: adjusted hazard ratio; IPCW: inverse probability of censoring weights; SNAE: serious non-AIDS event; 95% CI: 95% confidence interval

*Variables other than adherence were included only to reduce confounding of the association of interest, being adherence and all SNAEs, and should not be interpreted causally.

**Table 3: Adjusted associations with first SNAE using inverse probability of censoring weights**

| **Characteristic*** | **aHR** | **Robust se** | **P-value** | **95% CI** |
| --- | --- | --- | --- | --- |
| **ADHERENCE LEVEL, >90%** | 0.641 | 0.241 | 0.065 | (0.400 - 1.027) |
| Age, years | 1.015 | 0.009 | 0.102 | (0.997 - 1.033) |
| Sex (Ref: Female) | 1 |  |  |  |
| Male | 1.232 | 0.235 | 0.376 | (0.777 - 1.953) |
| HIV viral load, log_10_ copies/mL | **3.670** | **0.127** | **<0.001** | **(2.859** - **4.712)** |
| Any comorbidity, yes | **1.572** | **0.190** | **0.018** | **(1.083** - **2.283)** |
| Body mass index (BMI), kg/m^2^ | 1.007 | 0.017 | 0.661 | (0.975 - 1.042) |
| Systolic blood pressure (mmHg) | 0.993 | 0.005 | 0.188 | (0.984 - 1.003) |
| Smoking status (Ref: Current) | 1 |  |  |  |
| Never smoked | **0.282** | **0.184** | **<0.001** | **(0.196** - **0.404)** |
| Former smoker | **0.384** | **0.186** | **<0.001** | **(0.267** - **0.552)** |
| Protease inhibitor (PI)-based regimen (Ref: No) | 1 |  |  |  |
| Yes | 1.246 | 0.163 | 0.177 | (0.905 - 1.716) |
| CD4-count, cells/µL | **0.998** | **0.001** | **<0.001** | **(0.998** - **0.999)** |
| Nadir CD4-count, cells/µL | 1.000 | 0.001 | 0.496 | (0.999 - 1.002) |

aHR: adjusted hazard ratio; Robust se: robust standard error; 95% CI: 95% confidence interval

*Variables other than adherence were included only to reduce confounding of the association of interest, being adherence and first SNAE, and should not be interpreted causally.

**SENSITIVITY ANALYSES – ASSOCIATION OF ADHERENCE WITH SNAEs**

| **Characteristic*** | **OR** | **SE** | **P-value** | **95% CI** |
| --- | --- | --- | --- | --- |
| **ADHERENCE LEVEL (REF: ≤90%)** | 1 |  |  |  |
| **>90%** | **0.141** | **0.886** | **0.027** | **(0.025 - 0.803)** |
| Age, years | **1.182** | **0.063** | **0.008** | **(1.045 - 1.338)** |
| Sex (Ref: Female) | 1 |  |  |  |
| Male | **0.143** | **0.782** | **0.013** | **(0.031 - 0.662)** |
| HIV viral load, log copies/mL | 0.213 | 1.800 | 0.391 | (0.006 - 7.264) |
| Body mass index (BMI), kg/m^2^ | 0.852 | 0.083 | 0.053 | (0.724 - 1.002) |
| Systolic blood pressure (mm/Hg) | 0.963 | 0.046 | 0.415 | (0.880 - 1.054) |
| Smoking status (Ref: Current) | 1 |  |  |  |
| Never smoked | 0.220 | 1.907 | 0.428 | (0.005 - 9.257) |
| Former smoker | 0.636 | 1.412 | 0.749 | (0.040 - 10.127) |
| Protease inhibitor (PI)-based regimen (Ref: No) | 1 |  |  |  |
| Yes | 5.567 | 0.909 | 0.059 | (0.938 - 33.040) |
| CD4 cell count, cells/µL | 1.000 | 0.001 | 0.608 | (0.998 - 1.001) |
| Nadir CD4 cell count, cells/µL | 1.000 | 0.002 | 0.885 | (0.997 - 1.004) |

**Table 4: Association of adherence and adjustment variables with the development of first SNAE using GEE**

GEE: generalized estimating equations; OR: odds ratio; SE: standard error; SNAE: serious non-AIDS event; 95% CI: 95% confidence interval

*Variables other than adherence were included only to reduce confounding of the association of interest, being adherence and first SNAE, and should not be interpreted causally.

| **Characteristic*** | **OR** | **SE** | **P-value** | **95% CI** |
| --- | --- | --- | --- | --- |
| **ADHERENCE LEVEL (REF: ≤90%)** | 1 |  |  |  |
| **>90%** | 0.189 | 0.974 | 0.087 | (0.028 - 1.271) |
| Age, years | **1.148** | **0.043** | **0.001** | **(1.055 - 1.249)** |
| Sex (Ref: Female) | 1 |  |  |  |
| Male | 0.284 | 1.047 | 0.230 | (0.037 - 2.215) |
| HIV viral load, log copies/mL | 0.442 | 1.589 | 0.607 | (0.020 - 9.946) |
| Body mass index (BMI), kg/m^2^ | 0.877 | 0.070 | 0.057 | (0.767 - 1.004) |
| Systolic blood pressure (mm/Hg) | 0.993 | 0.034 | 0.837 | (0.929 - 1.061) |
| Smoking status (Ref: Current) | 1 |  |  |  |
| Never smoked | 0.704 | 1.462 | 0.810 | (0.040 - 12.359) |
| Former smoker | 1.282 | 1.222 | 0.839 | (0.117 - 14.057) |
| Protease inhibitor (PI)-based regimen (Ref: No) | 1 |  |  |  |
| Yes | 2.146 | 0.916 | 0.404 | (0.356 - 12.914) |
| CD4 cell count, cells/µL | 1.002 | 0.002 | 0.357 | (0.998 - 1.005) |
| Nadir CD4 cell count, cells/µL | 0.993 | 0.034 | 0.837 | (0.929 - 1.061) |

**Table 5:** **Association of adherence and adjustment variables with the development of all SNAEs using GEE**

GEE: generalized estimating equations; OR: odds ratio; SE: standard error; SNAE: serious non-AIDS event; 95% CI: 95% confidence interval

*Variables other than adherence were included only to reduce confounding of the association of interest, being adherence and all SNAEs, and should not be interpreted causally.

| **Characteristic*** | **aHR** | **Robust se** | **P-value** | **95% CI** |
| --- | --- | --- | --- | --- |
| **ADHERENCE LEVEL (REF: ≤90%)** | 1 |  |  |  |
| **>90%** | 0.641 | 0.301 | 0.141 | (0.356 - 1.158) |
| Age, years | 1.009 | 0.011 | 0.444 | (0.987 - 1.031) |
| Sex (Ref: Female) | 1 |  |  |  |
| Male | 1.440 | 0.284 | 0.199 | (0.826 - 2.511) |
| HIV viral load, log copies/mL | 1.272 | 0.247 | 0.330 | (0.784 - 2.065) |
| Body mass index (BMI), kg/m^2^ | 0.985 | 0.028 | 0.586 | (0.933 - 1.040) |
| Systolic blood pressure (mm/Hg) | 1.004 | 0.007 | 0.586 | (0.990 - 1.018) |
| Smoking status (Ref: Current) | 1 |  |  |  |
| Never smoked | **0.324** | **0.263** | **<0.001** | **(0.193 - 0.543)** |
| Former smoker | **0.313** | **0.240** | **<0.001** | **(0.196 - 0.500)** |
| Protease inhibitor (PI)-based regimen (Ref: No) | 1 |  |  |  |
| Yes | **1.684** | **0.250** | **0.037** | **(1.031 - 2.752)** |
| CD4 cell count, cells/µL | **0.998** | **0.001** | **0.010** | **(0.997 - 0.999)** |
| Nadir CD4 cell count, cells/µL | 1.001 | 0.001 | 0.238 | (0.999 - 1.002) |

**Table 6:** **Association of adherence and adjustment variables with the development of all SNAEs using IPCW in participants who remained virally suppressed <50 copies/mL for the duration of follow-up (147 SNAE events, n=1,984)**

aHR: adjusted hazard ratio; IPCW: inverse probability of censoring weights; SNAE: serious non-AIDS event; 95% CI: 95% confidence interval

*Variables other than adherence were included only to reduce confounding of the association of interest, being adherence and all SNAEs, and should not be interpreted causally.

| **Characteristic*** | **aHR** | **Robust se** | **P-value** | **95% CI** |
| --- | --- | --- | --- | --- |
| **ADHERENCE LEVEL (REF: ≤90%)** | 1 |  |  |  |
| **>90%** | 0.673 | 0.296 | 0.181 | (0.377 - 1.202) |
| Age, years | 1.015 | 0.011 | 0.148 | (0.995 - 1.037) |
| Sex (Ref: Female) | 1 |  |  |  |
| Male | 1.325 | 0.279 | 0.312 | (0.768 - 2.288) |
| HIV viral load, log copies/mL | 1.271 | 0.234 | 0.305 | (0.803 - 2.012) |
| Body mass index (BMI), kg/m^2^ | 0.994 | 0.023 | 0.805 | (0.950 - 1.040) |
| Systolic blood pressure (mm/Hg) | 0.997 | 0.006 | 0.571 | (0.985 - 1.009) |
| Smoking status (Ref: Current) | 1 |  |  |  |
| Never smoked | **0.263** | **0.231** | **<0.001** | **(0.168 - 0.413)** |
| Former smoker | **0.324** | **0.229** | **<0.001** | **(0.207 - 0.508)** |
| Protease inhibitor (PI)-based regimen (Ref: No) | 1 |  |  |  |
| Yes | 0.673 | 0.296 | 0.181 | (0.377 - 1.202) |
| CD4 cell count, cells/µL | 1.001 | 0.001 | 0.405 | (0.999 - 1.002) |
| Nadir CD4 cell count, cells/µL | 1.242 | 0.202 | 0.283 | (0.836 - 1.846) |

**Table 7:** **Association of adherence and adjustment variables with the development of first SNAE using IPCW in participants who remained virally suppressed <50 copies/mL for the duration of follow-up (135 SNAE events, n=1,997)**

aHR: adjusted hazard ratio; IPCW: inverse probability of censoring weights; SNAE: serious non-AIDS event; 95% CI: 95% confidence interval

*Variables other than adherence were included only to reduce confounding of the association of interest, being adherence and first SNAE, and should not be interpreted causally.
